# Insight to Nanoparticle Size Analysis—Novel and Convenient Image Analysis Method Versus Conventional Techniques

**DOI:** 10.1186/s11671-016-1391-z

**Published:** 2016-03-31

**Authors:** Minnamari Vippola, Masi Valkonen, Essi Sarlin, Mari Honkanen, Heikki Huttunen

**Affiliations:** Department of Materials Science, Tampere University of Technology, P.O. Box 589, 33101 Tampere, Finland; Department of Signal Processing, Tampere University of Technology, P.O. Box 553, 33101 Tampere, Finland

**Keywords:** Nanoparticles, Particle size analysis, TEM, Image analysis, SAXS, WAXS, Segmentation, Snake model, Balloon force

## Abstract

The aim of this paper is to introduce a new image analysis program “Nanoannotator” particularly developed for analyzing individual nanoparticles in transmission electron microscopy images. This paper describes the usefulness and efficiency of the program when analyzing nanoparticles, and at the same time, we compare it to more conventional nanoparticle analysis techniques. The techniques which we are concentrating here are transmission electron microscopy (TEM) linked with different image analysis methods and X-ray diffraction techniques. The developed program appeared as a good supplement to the field of particle analysis techniques, since the traditional image analysis programs suffer from the inability to separate the individual particles from agglomerates in the TEM images. The program is more efficient, and it offers more detailed morphological information of the particles than the manual technique. However, particle shapes that are very different from spherical proved to be problematic also for the novel program. When compared to X-ray techniques, the main advantage of the small-angle X-ray scattering (SAXS) method is the average data it provides from a very large amount of particles. However, the SAXS method does not provide any data about the shape or appearance of the sample.

## Background

Nowadays, nanoparticles are widely studied and used while their nanometer-scale size introduces such properties in them, which can differ significantly from those of the corresponding bulk material. Nanoparticles can be used in wide variety of applications where they can be considered for example as chemically inert additives like fillers in novel composite materials and high-refractive index and UV absorbing pigments in cosmetics and other consumer products or as chemically active particles in biomedical, in catalytic and in biotechnological use and like drug delivery agents for pharmaceutical industry [1–3]. Nanoparticles have unique properties which directly correlate to their size, shape, and size distribution, and therefore, to ensure the full exploitation of their properties, it is important to be able to measure these features efficiently and accurately. In recent years also huge efforts for estimating nanoparticles and related products health effects have been made [1, 3–5]. In order to provide reproducible nanoparticle characteristics for nanosafety studies and for compliance of nanoregulations, a precise determination of nanoparticle size and size distribution is crucial. Examples of methods used to characterize nanoparticles both for technical and academic use are transmission electron microscopy (TEM), scanning electron microscopy (SEM), scanning transmission electron microscopy (STEM), small-angle X-ray scattering (SAXS), wide-angle X-ray scattering (WAXS), atomic force microscopy (AFM), dynamic light scattering (DLS), differential mobility analysis (DMA), and time-of-flight secondary ion mass spectroscopy (TOF-SIMS) [2, 6–11]. Typically, different methods are seen as complementary techniques and they are recommended to be used together. Many of these methods mentioned above are different in terms of suitability to certain sized particles, easiness to use, time required, and other characteristics possible to obtain simultaneously.

In this paper, we are concentrating on transmission electron microscopy (TEM) methods into which different image analysis techniques are linked and on X-ray diffraction (SAXS and WAXS) techniques. The novelty of this paper is our new image analysis program “Nanoannotator”. This paper describes its usefulness and efficiency when analyzing nanoparticles, and at the same time, we compare it to conventional nanoparticle analysis techniques.

### Description of the Developed Image Analysis Software Nanoannotator

TEM images provide a visualization of the studied nanoparticles and thus essential information not only about the size, but also on other characteristics of the particles. However, the size distribution measurement from TEM images is typically a challenging task due to the tendency of nanoparticles to accumulate together on TEM grid. Manual measurement is a widely used approach, but is limited by a large variance between subjects and limited throughput. On the other hand, the ability of image processing algorithms to separate particles that are in contact with each other is often limited, as well. Thus, the problem grows around the topic of identifying individual particles and their shape from agglomerates. Once this task has been solved, the characteristics, such as the size, orientation, or shape factor of individual particles are easier to resolve.

An image analysis framework for studying the primary nanoparticle size distribution was developed. The particles are modeled using an active shape model [12]: the method fits an active contour to the edge map of a TEM image, and it is able to identify individual nanoparticles and to define their size and shape parameters.

The image analysis pipeline consists of three steps: center point approximation, particle segmentation, and parameter computation. Center point approximation aims to extract the center point of each individual particle in the image, thus resulting in a set of estimated coordinates of all particles present in the image. The second step finds a local homogeneous area around the center point of each particle. As a result, we will have an exact map or the area occupied by each particle. After all particles and their shapes have been separated, the final step is to compute any area- or shape-based quantities from the mutual arrangement of the pixels in a segment.

The proposed method partitions first image roughly into particle region and background region using Otsu’s thresholding. The first step begins by a rough segmentation of the image into foreground and background based on local brightness. For this purpose, we use the well-known Otsu segmentation [13], which finds a gray level minimizing the sum of variances of the foreground and background partitions. The result of the initial segmentation step gives a preliminary separation of the particles (dark) from the background (bright). The centroids of the particles are then located using the distance transformation, where the distance to the background region is computed for each foreground (particle) pixel. Since distance transform is known to result in high values close to particle centers and low values near borders, we can define the centroids as the locations of the local maxima in the transform.

The center points are needed in the second step where enclosed contour ***v***(*s*) = (*x*(*s*), *y*(*s*)) is moved within the image, and its position is updated iteratively into the direction of lowest energy. Here, the contour *v*(*s*) needs to be initialized before iterations can be run. In the case of round particles, one approach to form the initial contours is to draw a circle around each center point and to set their radii slightly larger than the distance from the center to the background region. Other initializations may be devised according to the shape characteristics of the analyzed particles.

The energy functional to be minimized in the traditional active contour algorithm [14] is defined as

1 $$ E={\displaystyle \underset{0}{\overset{1}{\int }}}{E}_{\mathrm{int}}\left(\boldsymbol{v}(s)\right) + {E}_{\mathrm{ext}}\left(\boldsymbol{v}(s)\right)ds, $$

where ${E}_{\mathrm{int}}=\frac{1}{2}\left(a{\left|{\boldsymbol{v}}^{\boldsymbol{\prime}}(s)\right|}^2 + b{\left|{\boldsymbol{v}}^{\boldsymbol{{\prime\prime}}}(s)\right|}^2\right)$ denotes to the internal energy and acts as regularizer and limits the possible states the snake can settle in. The first term of *E*_int_ (integral of the first derivative) restricts the length of the snake, and the second term (integral of the second derivative) discourages it from forming sharp corners. On the other hand, the external energy term *E*_ext_ pulls the contour towards certain gray values, edges, and line segment terminations. More specifically, we define and *E*_ext_ = *w*_line_*E*_line_ + *w*_edge_*E*_edge_ + *w*_term_*E*_term_ with *E*_line_, *E*_edge_, *E*_term_ denoting the strengths of a line, edge, and corners and terminations within the contour. Moreover, the coefficients *a*, *b*, *w*_line_, *w*_edge_, and *w*_term_ are model hyperparameters that can be used for tuning the algorithm to be attracted towards any of the mentioned features in an image. Once the particle segments have been extracted, various measures are calculated from the obtained segments. These parameters are listed in Table 1.

**Table 1:** Parameters computed for each particle segment and their descriptions

Parameter	Description
Area	The number of pixels in a region
Centroid	The center of pixel mass
Solidity	The ratio of shared pixels between the segment and its convex hull
Perimeter	The length of the resulting active contour around the studied particle.
Major axis length	Major axis length of an ellipse with the same second central moment as the segmented object
Minor axis length	Major axis length of an ellipse with the same second central moment as the segmented object
Orientation	Major axis orientation of an ellipse with the same second central moment as the segmented object
Equivalent diameter	Diameter of a circle that has the same area as the particle
Circularity	Ratio of shared pixels between the segment and its convex hull
Aspect ratio	The ratio of major and minor axis (see above)

In addition to the internal and external forces, the active contour model can be extended by including a balloon force *λ* that acts perpendicular to the contour. Depending on initialization of *λ*, the force inflates *(λ > 0)* or deflates *(λ < 0)* the contour on each iteration. It improves the original model by allowing the contour to be initialized far from the desired particle contour, which is beneficial since the framework relies on automatic contour initialization [15].

## Methods

In this study, the size distribution of three nanomaterials was studied. The studied materials were silver nanoparticles, iron oxide whiskers, and graphite nanoparticles. These materials were selected since they represent different extremes in terms of factors affecting the particle size analysis, as described in the next chapter. Silver nanoparticles from NANOGAP s.a. (Spain) have a product name of NGAP NP Ag-2103 and they are a mixture of quasi-spherical and rod-like particles with the mean particle size of 40–55 nm [16]. Iron oxide whiskers from Nanostructured & Amorphous Materials Inc., (USA) have a product name of α Fe2O3 fiber with the fiber diameter of 40–150 nm and fiber length of 250–600 nm [17]. Graphite nanoparticles from SkySpring Nanomaterials Inc., (USA) have a product name of graphite nanoparticles #0520BX with spherical particle morphology and the average particle size of 3–4 nm [18].

The size distribution of the three nanomaterials was studied by image analysis based on transmission electron microscopy (TEM) images and by small- and wide-angle X-ray scattering (SAXS/WAXS). JEOL JEM 2010 transmission electron microscope was used to study the nanomaterials. The samples were prepared by slightly crushing the nanomaterial powder between laboratory glass slides, mixing the powder with ethanol and by dropping the dispersion on the copper TEM grid with a holey carbon film. Similar imaging conditions were used for all nanoparticles (acceleration voltage 200 kV, large objective aperture).

Three different image analysis methods were compared: traditional manual image analysis, an open source image processing program ImageJ (http://imagej.net) and the MATLAB-based image analysis program Nanoannotator described in the previous chapter. The details of the image analysis practices are described together with the results. Particle size distribution by number (*D*_*n*_) was determined by the image analysis methods. The results were compared to the volume-weighted particle-size distributions (*D*_*v*_) defined by small-angle X-ray scattering (SAXS) method. Although in general, size distributions by number and by volume are not comparable, these two distributions result in similar average and most frequent particle sizes if the size distribution is relatively narrow [10]. In addition, to compare the particle size and crystallite size of three nanomaterials, also wide-angle X-ray scattering (WAXS) measurements were performed.

The SAXS and WAXS measurements were done by Panalytical Empyrean Multipurpose Diffractometer with Cu Kα X-ray source (*λ* = 0.15418 nm) and a solid-state detector (PIXcel3D). For SAXS measurements, a focusing mirror for Cu radiation was used. Mylar foil was applied on both sides of the sample, and a double Mylar film was used as the background sample. The studied angle range 2*θ* was −0.1…5° with a step size of 0.01° and step duration of 3 s. The EasySAXS software (version 2.0a) was used to derive the volume-weighted particle-size distributions (*D*_*v*_) by indirect Fourier-transformation. An average crystallite size for nanomaterials was determined from WAXS patterns with the aid of the HighScore Plus software (version 3.0.5) and based on the well-known Scherrer equation. Before that, phases were identified by using the database (PDF-4+ 2014) from International Centre for Diffraction Data (ICDD).

## Results

**Fig. 1:**
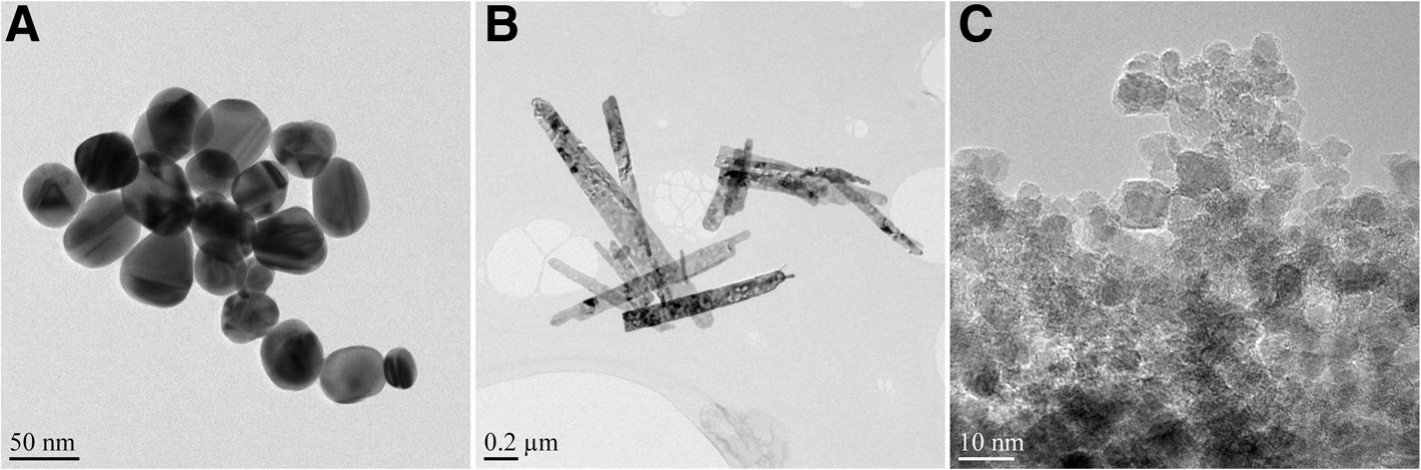
Silver nanoparticles (**a**), iron oxide whiskers (**b**), and graphite nanoparticles (**c**) used in the study. Note the different scale bars in the images

Examples of the studied materials are shown in Fig. 1. The silver and the graphite nanoparticles are quite circular whereas the iron oxide whiskers are needle-like particles. The silver nanoparticles are larger, and the individual particles are much easier to distinguish by the naked eye from a TEM image when compared to the graphite. Instead, the graphite nanoparticles tend to agglomerate, and their small size and poorer contrast due to lighter weight make it difficult to perceive the contours of the particles from the TEM images even by the naked eye. Also, the individual iron oxide whiskers were occasionally difficult to separate from a TEM image due to their variable size and surface topography. In the following sections, the methods, the particle size distribution results and the practicality of the different image analysis methods, and the SAXS technique are described.

### Manual Image Analysis

**Fig. 2:**
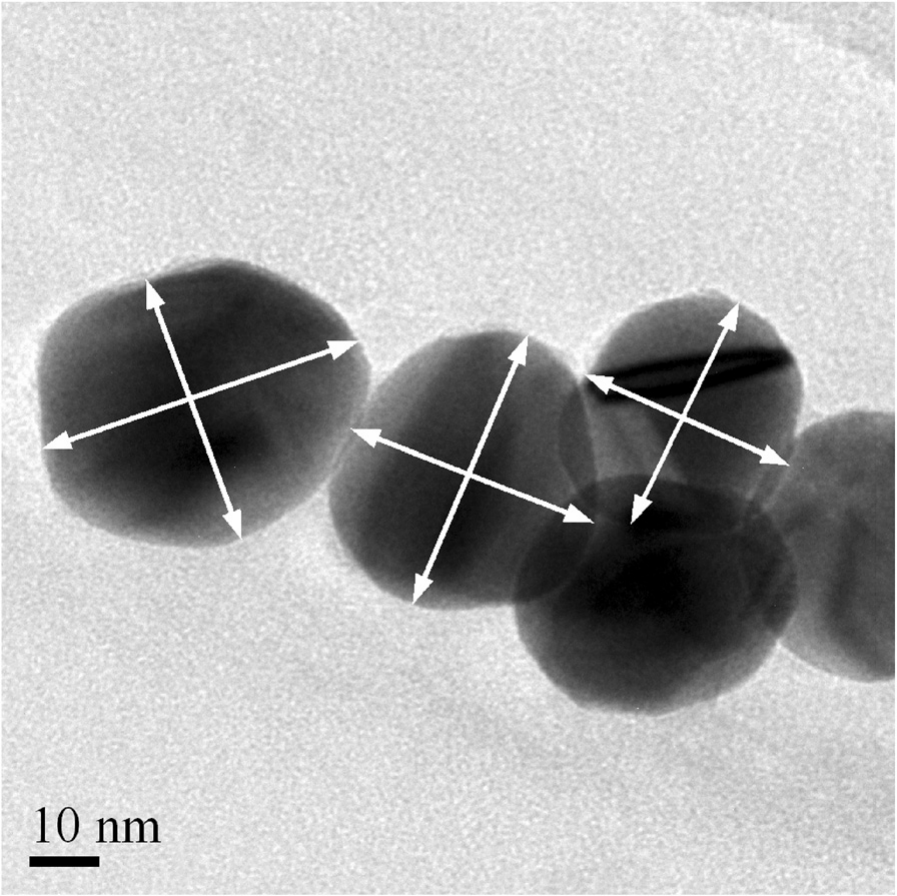
The manual image analysis was done by measuring two perpendicular diameters from particles

The manual image analysis was done by measuring two perpendicular diameters from the particle (Fig. 2) with DigitalMicrograph software (version 1.81.78) from Gatan Inc. and by evaluating the equivalent diameter of the particle size by the average of the measured dimensions. The manual image analysis was done for the silver nanoparticles, and it was regarded as the reference method for other ones in terms of accuracy. The results of the manual image analysis together with the results of other techniques are shown in Table 2. To define the most frequent value of the measured data, the results were grouped into 2-nm scale groups.

**Table 2:** The particle size results obtained by different methods. The relative standard deviations values shall be compared only within one sample type, since it is dependent on the particle size

	The size reported by manufacturer		Manual image analysis (*D*_*n*_)	Nanoannotator program (*D*_*n*_)		SAXS (*D*_*v*_)
Silver nanoparticles (diameter)						
Average size	40–55 nm [16]		44.0 nm	50.1 nm		43.5 nm
Most frequent value	–		42–44 nm	42–44 nm		35.0 nm
Relative standard deviation	–		26.0 %	30.2 %		48.8 %
Iron oxide whiskers (length and width)						
Average size	40–150 nm [17]	250–600 nm [17]	–	127.5 nm	939.0 nm	40.9 nm^a^
Most frequent value	–	–	–	117–127 nm	1050–1060 nm	29.0 nm^a^
Relative standard deviation	–	–	–	41.1 %	41.5 %	54.0 %^a^
Graphite (diameter)						
Average size	3–4 nm [18]		–	–		4.6 nm
Most frequent value	–		–	–		3.0 nm
Relative standard deviation	–		–	–		75.9 %

^a^In the case of whiskers, the default spherical shape of particles of the SAXS software is false

### “ImageJ” Open Source Image Analysis Program

**Fig. 3:**
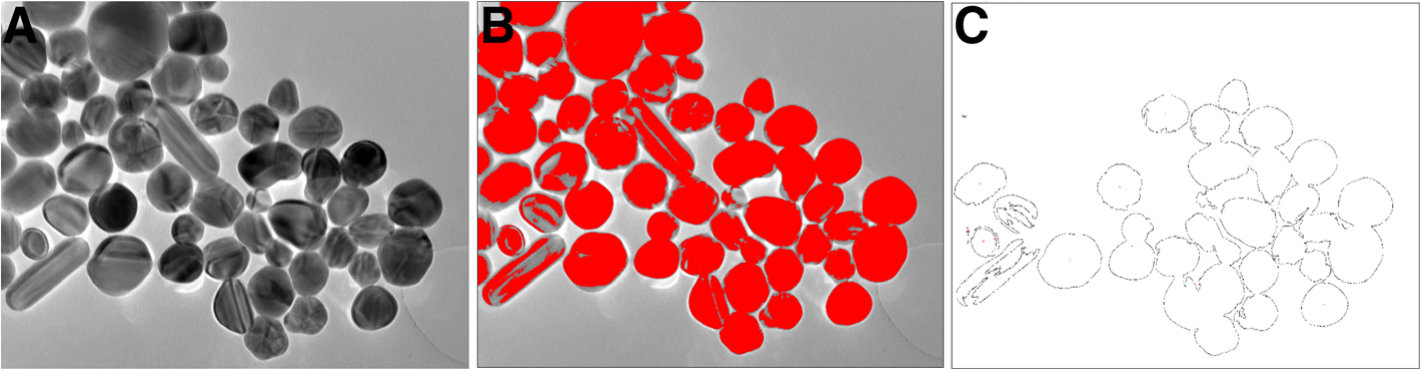
The particle analysis of silver nanoparticles is problematic by traditional image analysis program: (**a**) the original TEM image, (**b**) the image threshold set into an optimum level to distinguish the background and the particles, (**c**) after optimal thresholding, the program does not distinguish the actual individual particles

Traditionally, the particle analysis from TEM images is based on the conversion of the grayscale (TEM) image into black and white image by thresholding. From the converted black and white images, the program identifies the outlines of the particles. If the particles are non-aggregating, each outline correspond the 2D projection of a particle and the particle characteristics, such as the particle area, perimeter, aspect ratio, or orientation, can be automatically calculated [19]. However, the tendency of nanoparticles to agglomerate makes this task difficult, since the individual particles are impossible to separate by this method, as illustrated for the silver nanoparticles in Fig. 3. The converted image (Fig. 3c) consists of particle agglomerates, and the contours of individual particles are lost. Also, the number of the detected particles is too low since the program excludes all particles which are partly outside the image. Thus, it was not possible to use the ImageJ in any of the cases of this study to evaluate the size distribution of the nanoparticles with an acceptable accuracy.

### Nanoannotator Image Analysis Program

**Fig. 4:**
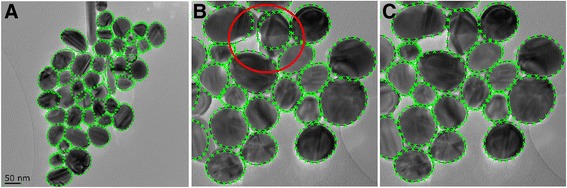
The particle analysis of silver nanoparticles by the developed program: (**a**) the automatically identified particles, (**b**) detail of an area requiring manual correction, (**c**) the same area after manual corrections

As described in the “Description of the developed image analysis software Nanoannotator” section, the developed program was specifically tuned for the silver nanoparticles. Thus, it was expected that the analysis of silver particles is straightforward and accurate. The program was able to identify the particles easily, and the automatic setting of the particle boundaries was accurate in most cases. The automatic particle identification and typical corrections required are exemplified in Fig. 4. Similar to any image analysis program, the particles partly outside the image were excluded. The characteristic used to describe the size of the particles was the equivalent diameter of the particles. The numerical results are shown in Table 1. The definition of the most frequent value of the obtained results was done similar as in the case of the manual image analysis results.

**Fig. 5:**
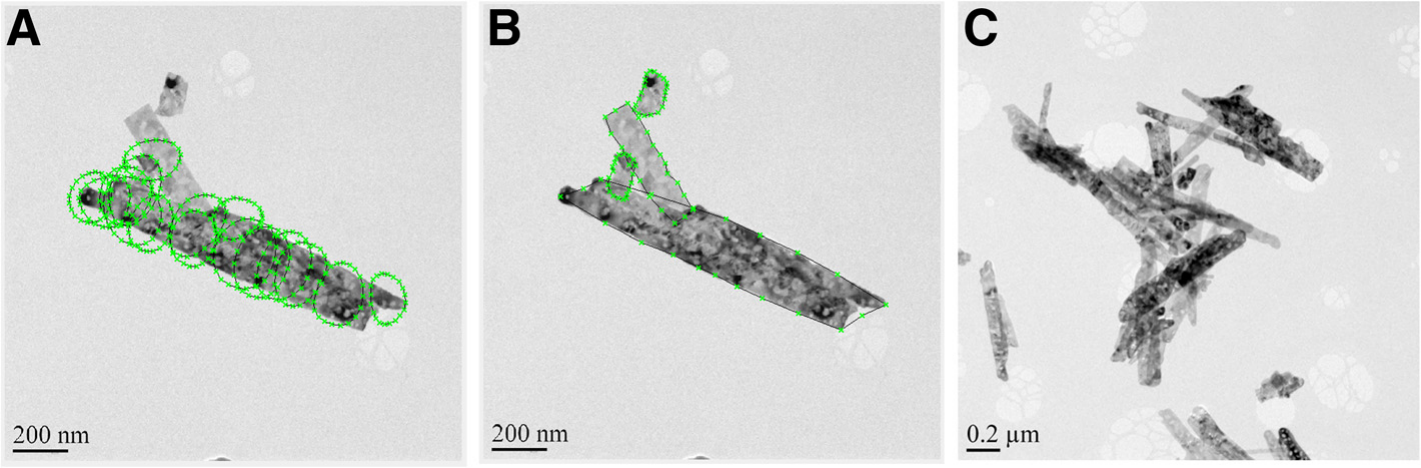
The particle analysis of iron oxide whiskers by the developed program: (**a**) automatically identified particles, (**b**) manually identified and shaped particle contours, (**c**) example of an area impossible to be analyzed even by the naked eye

The automatic analysis of the iron oxide whiskers by the Nanoannotator program did not succeed as well as for the silver nanoparticles. The presumption of the program that the particle shape is close to circular made the automatic identification of the whiskers impossible, as shown in Fig. 5a. Thus, the identification of the particles and the particle shape adjustment had to be made manually (Fig. 5b), which decreased the efficiency of the method. In addition, the agglomerated whiskers were partly impossible to distinguish even by the naked eye (Fig. 5c), and as a consequence, a lot of the particles in the TEM images could not be used in the image analysis. The characteristic used to describe the size of the whiskers was the major and the minor axes, since those were regarded as the most comparative values to the size reported by the manufacturer (length and width of the whisker). To define the most frequent value, a broader scale was used in grouping the results when compared to the silver nanoparticles due to the larger size of the whiskers.

**Fig. 6:**
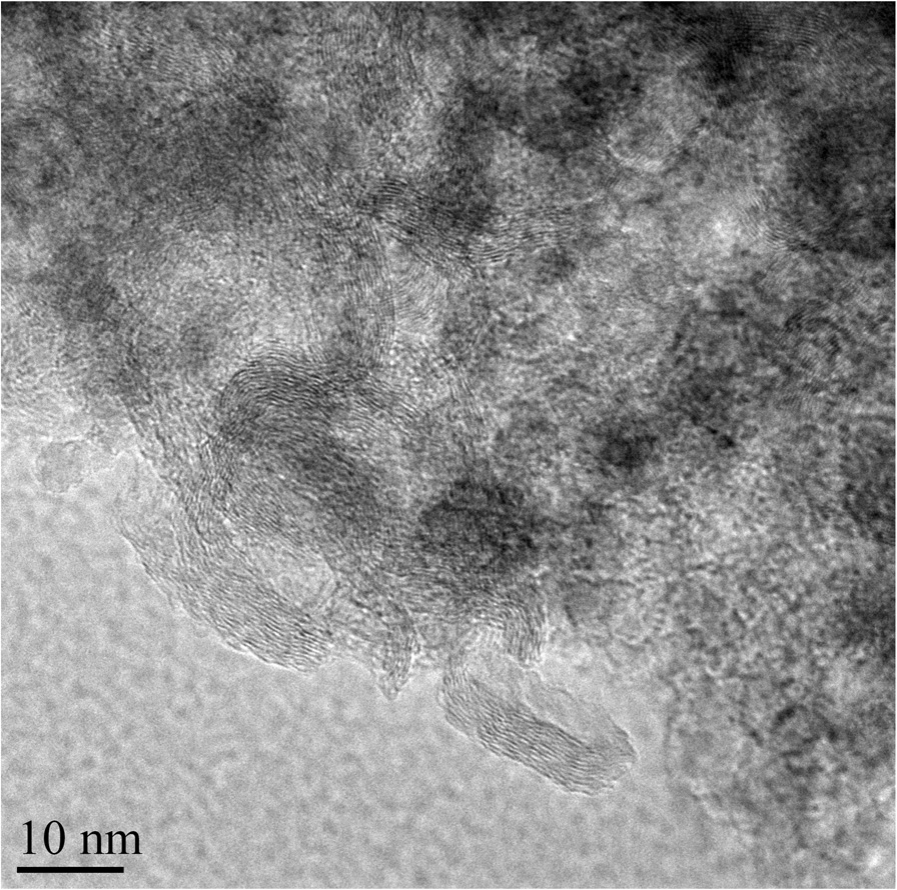
Graphite nanoparticles: individual particles are difficult to identify from the agglomerates and the contrast formation due to the inner structure of the particles complicates the analysis further

The small and roundish graphite nanoparticles were also problematic to analyze with the Nanoannotator program: the tendency of the particles to agglomerate and the ability of TEM to resolve the inner structure of the graphite particles (Fig. 6) make image analysis and evaluation of its success almost impossible. Thus, the graphite particles were not analyzed at all with image analysis techniques.

### SAXS and WAXS Measurements

The SAXS measurement is a relatively straightforward method to analyze nanoparticles. The sample preparation with Mylar film takes only few minutes per sample, and the actual measurement duration is minutes (in this study 25 minutes, but also shorter runs could be used). The particle size data obtained by different methods are summarized in Table 1.

**Fig. 7:**
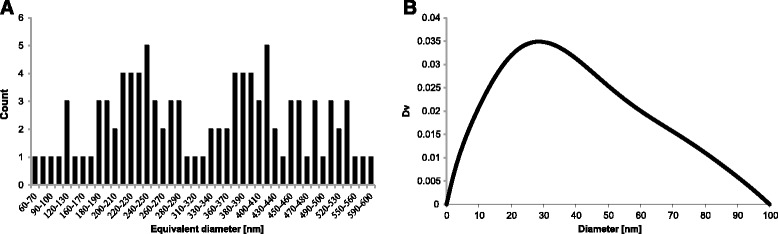
The size distribution of iron whiskers (**a**) by number as a result of image analysis method and (**b**) by volume as a result of SAXS analysis

The possible problem of the SAXS method lies in analyzing the obtained data. In general, SAXS analysis methods presume a constant shape for the nanoparticles [8], and in SAXS software, it is typically spherical. This is also the case for the EasySAXS software (version 2.0a) where the calculation of the volume distributions is available only for spherical nanoparticles. Thus, the default assumption of spherical shape of particles is false and causes error in the results. The differences in the SAXS and image analysis results are illustrated in Fig. 7 in which the Nanoannotator results (average equivalent diameter) and SAXS results (diameter) are compared. The binary distribution of the image analysis technique results reflects the needle-like shape of the particles (whisker length and width) whereas the presumption of spherical particles loses the effect from the SAXS results. For shapes which are different from spheres, one can do simulations and fitting in SAXS software, but it will provide only average values of the dimensions.

**Fig. 8:**
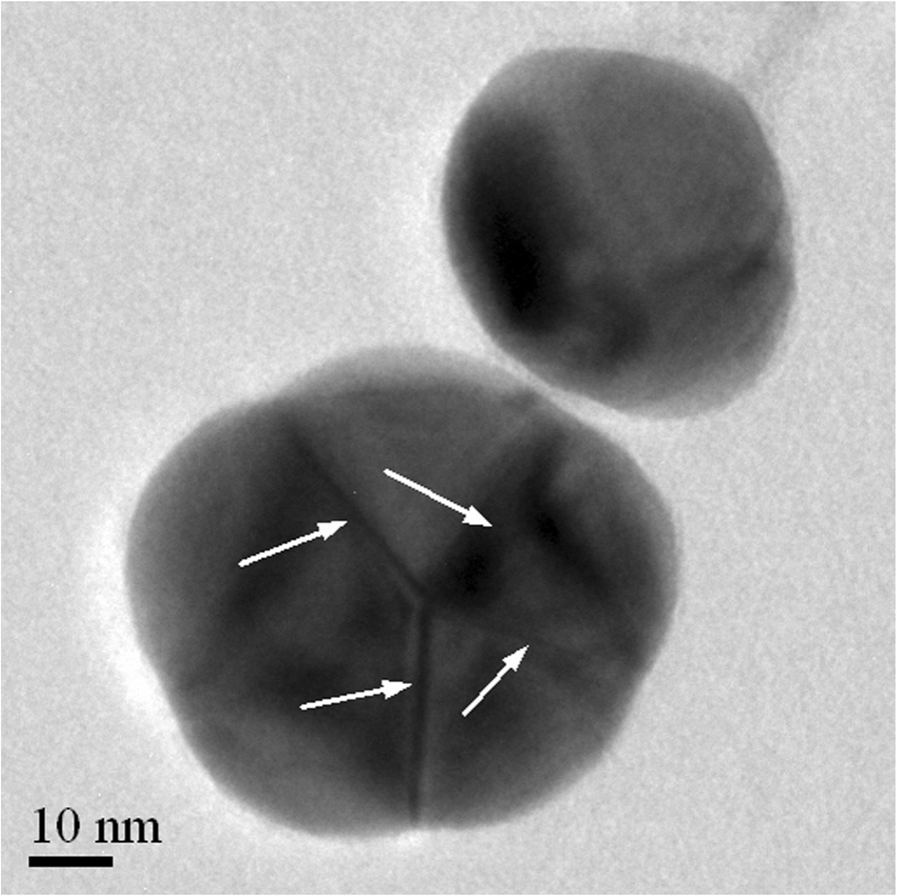
Silver nanoparticles, crystallite boundaries marked with *arrows* on the larger particle

The average crystallite sizes of nanomaterials were determined to compare them to the average particle sizes. The determination was made from WAXS patterns with the aid of the HighScore Plus software (version 3.0.5) and based on the well-known Scherrer equation with a typical crystallite shape factor value 0.9. This value is valid for spherical-shaped crystallites. Other possible shapes in the software are cubes, tetrahedrons, and octahedrons. Based on the measurements, the average crystallite size of silver nanoparticles is 20.5 nm calculated from the Ag (111) peak at 38.3° (2*θ*). The determined average diameter for the silver particles is 44–50 nm (Table 1) indicating that the larger particles consist of several crystallites which is also observed by TEM (Fig. 8). The average crystallite size of iron oxide whiskers is 18.8 nm calculated from the Fe_2_O_3_ (104) peak at 33.2° (2*θ*). The average length for the iron oxide whiskers was 128 nm and width 939 nm determined by Nanoannotator (Table 1). Remarkable difference between the average particle and crystallite sizes indicates that the whiskers also consist of several crystallites. However, individual crystallites in the strongly agglomerated whiskers are hard to recognize even by TEM. The average crystallite size of graphite is 3.6 nm calculated from graphite (100) peak at 42.5° (2*θ*). In the case of graphite, the average particle size and crystal size are similar indicating that the graphite nanoparticles are mainly single crystals.

### The Efficiency of the Different Methods

The efficiency of the different methods was evaluated by recording the time required for the particle analysis in each case and the results are shown in Table 3. In the case of TEM image analysis methods, the recorded time consisted of the time required to prepare the sample, take the images, and the time of the image analysis. Similarly, the efficiency of SAXS method was evaluated by the time required for sample preparation, data acquisition, and data analysis.

**Table 3:** The efficiency of the different methods is estimated by comparing the time required in each case to achieve the results

Sample	TEM imaging and manual image analysis	Number of analyzed particles	TEM imaging and analysis with Nanoannotator	Number of analyzed particles	SAXS data acquisition and analysis^a^
Ag	0.7 min/particle	100	0.2 min/particle	400	~30 min
Fe2O3	–	–	0.9 min/particle	100	
Graphite	–	–	–	–	

^a^The number of the analyzed particles in SAXS measurements is very large when compared to the image analysis techniques. Also, the time required is highly dependent on the SAXS acquisition time

In the case of silver nanoparticles, the accuracy of the automatic particle recognition of the Nanoannotator program was so good that the efficiency was three times better than in the manual image analysis. However, the challenging shape of the iron oxide whiskers decreased the efficiency of the Nanoannotator program, and it did not offer any advantage when compared with manual analysis. It is assumed that the manual analysis efficiency would be the same for different particle shapes. It should be noted that the described measurement durations do not represent any absolute values for these methods since the time is dependent on the operator and on the specimen, among other things.

## Discussion

The studied three different nanomaterials exemplified well how significant the effect of the material itself is on the practicality and efficiency of the used characterization method. The composition of the material, the particle shape and size, and the crystal size versus the particle size define the mass-thickness contrast of the TEM image. If high magnifications are used for small particles, also the inner structure of the particles can become visible, as for the graphite in our case. In this study, the focus was rather on image analysis than on optimizing the imaging conditions for each nanomaterial separately. In optimal case for particle size analysis, mass-thickness contrast solely provides best images. However, especially light and small particles, like graphite, require imaging conditions in which other contrast mechanisms are also visible. For example, increasing the objective aperture size decreases diffraction contrast in the image, but simultaneously, the effect of phase contrast is increased. Optimizing imaging conditions for a certain material can offer advantages for the particle size analysis, but in the end, the different contrast mechanisms always have some contribution to the formation of TEM images. In addition, the tendency of the material to agglomerate affects to the easiness to interpret the TEM images. The typical performance of an average observer can be estimated by the Rose criterion [20] which states that if the change in signal of an image exceeds the noise by a factor of five (*ΔS > 5 N*), it is visible to the human eye. So, if the outlines of the overlapping particles or the inner structure or topography of the particles itself cause high noise level, it can be very difficult to distinguish the particles. In these cases, the determination of the size distribution by image analysis techniques is not possible. Conventional TEM image analysis techniques require good dispersion of nanoparticles which needs a lot of resources and time for sample preparation [8, 9]. However, the ability of Nanoannotator to resolve individual particles when they are touching each other or even overlapping to some extent enables faster and straightforward sample preparation.

If the particle outlines are visible to the human eye from the image, different image analysis methods are applicable or at least the accuracy of the results can be evaluated. The general advantage of image analysis techniques is that in addition to the size distribution data, it provides other particle characteristics in the form of visual image of the particle. Also, the acquisition of elemental data by energy-dispersive X-ray spectroscopy (EDS) or crystallinity information by diffraction patterns is possible simultaneously to the imaging. The developed Nanoannotator program is a good supplement to the field of particle analysis techniques, since the traditional image analysis programs suffer from the inability to separate the individual particles from agglomerates and are thus useless. It is three times more efficient, and it offers a lot more detailed information of the particles than the manual technique. However, particle shapes very different from spherical proved to be problematic also for the Nanoannotator program and no benefit was achieved by its use. However, the program could be modified for different shaped particles and a similar efficiency as seen for the silver nanoparticles could be achieved. If the task is to measure the size distribution of agglomerates, which is a realistic case, for example, when evaluating the safety of some nanomaterials in air, also the traditional programs are practical. The only limitation of the manual image analysis is the resources it takes and the limitedness of the obtained data. It gives accurate results on particle dimensions and typically even the cases which are problematic to the image analysis methods are possible to analyze visually.

The advantage of the SAXS method is the average data it provides from a very large amount of particles. Also, the acquisition of the WAXS data from the same sample does not significantly increase the measurement time, but it provides additional data of the composition and crystal structure and size of the sample. However, the SAXS method does not provide any data about the shape or appearance of the sample. Since the shape data is required or at least helpful when analyzing the SAXS data, this method typically requires a complementary method. For small spherical particles, as for graphite, the SAXS method is optimal whereas the image analysis methods are problematic. However, for non-spherical particles, the SAXS data can be only compared to the simulated curves and the accuracy of the measurement decreases.

For non-uniform samples in which particle size or shape has a lot of variations, none of the aforementioned methods is suitable. However, the recent development of the computer technology offers new possibilities also for accurate and efficient image analysis techniques: the combination of excellent ability of the human eye to distinguish the particle outlines, touch screen, and stylus pen could offer the advantages of manual image analysis (accuracy) and image analysis programs (detailed size and shape information). In general, it can be stated that the nanoparticle analysis method has to be chosen according to the requirements of the case in question and the comparative studies between different nanomaterials is difficult.

## Conclusions

A novel image analysis program Nanoannotator used for nanoparticle analysis from transmission electron microscopy images was introduced, and its usefulness and efficiency was evaluated with promising results. This new image analysis program proved out to be a good supplement to the field of particle analysis techniques while having several advantages over the conventional analysis methods. The developed Nanoannotator program can separate the individual particles from agglomerates, it is more efficient in time and it offers detailed information from the particles. Nevertheless, Nanoannotator program does not solve all the challenges there are within the comprehensive nanoparticle analysis. Like when the particle shapes are very different from spherical ones, there are still issues to be solved for having them analyzed efficiently. In general, it can be stated that the nanoparticle analysis method has to be chosen according to the requirements for what the analysis is used, and an approach of using more than one nanoparticle analysis method should be favored in order to gain comprehensive data on size, size distribution, and shape of nanoparticles.
